# Botulinum Toxin Type A in Trigeminal Neuralgia Treatment: A Case Series and Literature Review

**DOI:** 10.7759/cureus.73389

**Published:** 2024-11-10

**Authors:** Rita F Santos, Manuel Machado, Margarida Ferro, Alexandre Camões-Barbosa

**Affiliations:** 1 Physical and Rehabilitation Medicine Department, Centro Hospitalar de Leiria, Leiria, PRT; 2 Neurology Department, Hospital de São José, Unidade Local de Saúde de São José, Lisboa, PRT; 3 Unidade de Neurofisiologia Clínica, Centro Hospitalar Universitário de Lisboa Central, Lisboa, PRT

**Keywords:** botulinum toxin, craniofacial pain, neuropathic pain, neurotoxin, trigeminal neuralgia

## Abstract

Trigeminal neuralgia (TN) stands as a common neuropathic pain disorder. Clinically, it manifests with episodes characterized by unilateral electric shock-like or knife-like pain that can involve one or more divisions of the fifth cranial nerve. Botulinum neurotoxin type A (BoNT-A) is a neurotoxin that has demonstrated analgesic effects in neuropathic pain and positive benefits in the treatment of refractory idiopathic TN. In this paper, we aim to describe the clinical outcomes and characteristics of a series of patients with TN who have undergone BoNT-A treatment within our clinical setting. Additionally, we aim to conduct a narrative review focusing on the use of BoNT-A in the management of TN. We present a case series of four patients diagnosed with idiopathic TN who underwent evaluation and treatment at our Neurotoxin Clinic from 2015 to 2023. BoNT-A was administered for the management of refractory idiopathic TN, and retrospective data collection was conducted. Patients had a confirmed diagnosis of idiopathic TN, determined through prior normal MRI and electrophysiological tests. The mean age was 46 years (range: 40-53 years), with TN onset occurring after the age of 40 years in all cases. The average time since TN onset was 11 years, and the average follow-up time was 6.25 years. Onabotulinumtoxin-A (ONA/A) was the BoNT-A formulation used, and the number of treatments during the follow-up period ranged from 12 to 37, with a mean of 23.75 injections. At six weeks post-injection, all patients underwent systematic reevaluation, assessing latency time, clinical response, and adverse reactions. Outcomes were quantified using a 0-10 cm Visual Analog Scale (VAS) for pain intensity and the mean frequency of pain attacks per day. Flexible injection intervals were adjusted based on each patient’s individual response duration. The mean improvement of pain severity on the VAS was 3.72 cm, and the mean reduction in the number of pain attacks per day was 77.32%. The average latency until the onset of treatment effect was 9.5 days, and the average duration of treatment effectiveness was 15.26 weeks. Adverse reactions were exhibited in two patients but with a low degree of severity. We also conducted a narrative review focusing on the use of BoNT-A in the management of TN. The review included randomized, double-blind, placebo-controlled trials (RCT) published between January 2012 and December 2023. Data from the four included RCTs favored the use of BoNT-A. Overall, attack frequency and pain severity were reduced one to two weeks after injections, and treatment demonstrated efficacy throughout the study follow-up until 12 weeks, as also happened in our case series. BoNT-A doses ranged from 25 U to 75 U. Based on the available evidence, BoNT-A seems to be effective and safe in the treatment of refractory idiopathic TN for up to 12 weeks. Repeated injections may be safe and maintain treatment effectiveness. The optimal dose and the duration of treatment effectiveness remain uncertain. Further studies are necessary to provide a more comprehensive understanding of BoNT-A in the treatment of TN.

## Introduction

Trigeminal neuralgia (TN) stands as a common neuropathic pain disorder with a lifetime prevalence ranging from 0.03% to 0.3% and an incidence that increases proportionally with age (4-29 per 100000 person-years) [1]. It exhibits a higher occurrence in women (F:M ratio of 3:1-2) and in 90% symptoms begin after the age of 40, with a mean onset age of 53 years [1,2].

Clinically, TN is marked by recurrent, paroxysmal, severe, and unilateral electric shock-like or knife-like pain episodes, lasting between a second to two minutes (rarely exceeding this timeframe), with an abrupt onset and termination, and involving one or more divisions of the fifth cranial nerve. These episodes can arise spontaneously or are precipitated by innocuous triggers such as cold weather, brushing teeth, or shaving. Idiopathic and classical TN may exhibit purely paroxysmal characteristics or present with concurrent persistent background continuous facial pain. Over time, the pain may intensify and become associated with mild autonomic symptoms like lacrimation and/or eye redness. Importantly, sensory abnormalities within the trigeminal distribution are typically absent during bedside testing, and in severe cases, facial muscle contractions on the affected side (tic douloureux) may occur [3,4].

The International Classification of Headache Disorders (ICHD-3, 2018) classifies TN into three types: (1) classical, (2) secondary, and (3) idiopathic [3,5]. For a diagnosis of classical TN, neurovascular compression, demonstrated through MRI or surgery, must be evident, showing displacement and/or atrophy of the trigeminal nerve, usually at the root entry zone, caused by an artery or vein. Secondary TN may result from inflammatory lesions like those in multiple sclerosis, space-occupying lesions such as a cerebellopontine angle tumor, or other causative factors. In idiopathic TN, neither electrophysiological tests nor MRI show significant abnormalities [1,3].

The primary approach for treating TN typically involves the use of one or more oral anticonvulsant drugs, in particular sodium channel blockers like carbamazepine and oxcarbazepine [1,6].

Nevertheless, approximately 25% to 50% of patients experience a reduced responsiveness to oral drug therapy demanding higher doses, which may lead to an increased risk of potential side effects. In cases where patients exhibit inadequate response or intolerance to oral drug treatment, other treatments can be sought, including surgery [5,6].

Surgical interventions for TN can be categorized into three types: (1) invasive, non-ablative procedures such as microvascular decompression; (2) invasive, ablative techniques like percutaneous balloon compression, percutaneous glycerol rhizotomy, and percutaneous radiofrequency rhizotomy; and (3) non-invasive ablative approaches, including stereotactic radiosurgery. However, all these surgical treatments are associated with various potential postoperative complications, with facial sensory changes being the most common complication, irrespective of the specific procedure undertaken [6].

Some studies have demonstrated the analgesic effects of botulinum neurotoxin type A (BoNT-A) in TN [7-9]. BoNT-A is a potent natural neurotoxin produced by Clostridium botulinum that blocks acetylcholine release at the neuromuscular junction by interfering with the soluble N-ethylmaleimide-sensitive factor attachment protein receptor (SNARE) complex, eventually leading to muscle relaxation. Currently, in both Europe and the USA, various formulations of BoNT-A are available, specifically abobotulinumtoxin-A, incobotulinumtoxin-A, and onabotulinumtoxin-A (ONA/A). These formulations find application in treating a range of conditions, encompassing spasticity and dystonia [10]. There is also published evidence supporting the analgesic effects of BoNT-A injections in several pain conditions, including central poststroke pain and spasticity-associated pain due to diverse etiologies (stroke, multiple sclerosis, or cerebral palsy, meningioma), hemiplegic shoulder pain, neuropathic pruritus, hemifacial spasm, and pain due to refractory temporomandibular disorders [11-17].

The analgesic effect of BoNT-A was initially attributed to its muscle relaxation properties. However, recent studies using BoNT-A in neuropathic pain models have confirmed that there is an analgesic effect independent of muscle relaxation [18]. This underscores the evolving understanding of BoNT-A's multifaceted mechanisms beyond its originally perceived muscle-relaxing function.

In this paper, we aim to describe the characteristics and clinical outcomes of a series of patients with TN who have undergone BoNT-A treatment within our clinical setting. Additionally, we aim to conduct a narrative review focusing on the use of BoNT-A in the management of TN. 

The review included randomized, double-blind, placebo-controlled trials (RCT) published between January 2012 and December 2023, in the English language. The primary information sources consulted were MEDLINE (PubMed) and the Cochrane Library.

## Case presentation

Case series

We present a case series of four patients diagnosed with idiopathic TN who underwent evaluation and treatment at our Neurotoxin Clinic (Hospital de São José, Unidade Local de Saúde de São José, Lisbon, Portugal), from 2015 to 2023.

BoNT-A was administered for the management of refractory idiopathic TN, and retrospective data collection was conducted. Patients selected for the case series met the following criteria: confirmed diagnosis of idiopathic TN determined through prior normal MRI and electrophysiological tests and previous ineffective treatment with pharmacological agents. Patient characteristics, including age, sex, time since TN onset, TN localization, and oral medication used, were collected and summarized in Table 1. ONA/A was the BoNT-A formulation used. The treatment protocol consisted of ONA/A subcutaneous injections given 1 cm apart from one another in the path of the trigeminal branch/branches involved, according to the patients’ descriptions of the painful area, in a “follow the pain” strategy. Each point was injected with 5 U/0.1 mL. Injected doses ranged from 30 U (six injection points) to 60 U (12 points). At six weeks post-injection, all patients underwent systematic reevaluation, assessing latency time, clinical response, and adverse reactions. Outcomes were quantified using a 0-10 cm Visual Analog Scale (VAS) for pain intensity and the mean frequency of pain attacks per day. Monthly reevaluation was maintained to continuously evaluate the patient's symptoms and the need for another cycle of injections with BonT-A. During the follow-up period, flexible injection intervals were adjusted based on each patient’s individual response duration. These results are compiled and presented in Table 1. 

**Table 1:** Case series: baseline characteristics, intervention, and outcomes ONA/A: onabotulinumtoxin-A; VAS, Visual Analog Scale; TN, trigeminal neuralgia; BoNT-A, botulinum neurotoxin type A

	Baseline							Follow-up						
Patient	Age	Sex	Time since TN onset/follow-up period in years	Localization	Medications currently used	Reported pain intensity during attacks before BoNT-A treatment (VAS)	Reported mean frequency of pain attacks per day before BoNT-A treatment	Type of BoNT-A used	Number of completed treatment cycles with BoNT-A	Dose, mean	Latency, mean (days)/ Effect duration mean (weeks)	Reported pain intensity during attacks after BoNT-A treatment (four-week assessment) (VAS)	Reported mean number of pain attacks per day after BoNT-A treatment	Adverse reactions (n, %)
1	53	M	14/4	V2+V3, right	Carbamazepine, pregabalin, amitriptyline	9.0 cm	50.0	ONA/A	12	60 U	11.00/14.25	5.5 cm	2.0	None
2	42	F	15/8	V2+V3, left	Carbamazepine	9.0 cm	20	ONA/A	34	50 U	8.10/11.90	7.2 cm	6	Mild xerostomia (n=7, 20.6%), mild pain (n=2, 5.9%)
3	40	F	8/8	V2+V3, left	Amitriptyline	6.5 cm	12	ONA/A	37	30 U	10.56/10.63	24.9 cm	5.6	Mild xerostomia (n=2, 5.4%), mild Pain (n=2, 2.7%), mild oral incontinence (n=6, 16.2%), mild facial paralysis (n=12, 32.4%)
4	49	F	7/5	V3, left	Oxcarbazepine	8 cm	100	ONA/A	12	45 U	8.45/24.27	0 cm	10	None

Patient 1

A 53-year-old male with a 14-year history of trigeminal neuralgia (TN), localized to the V2/V3 divisions on the right hemiface. Medications currently used were carbamazepine, pregabalin, and amitriptyline. He had a four-year follow-up period during which he received 12 cycles of treatment with ONA/A. He reported a significant improvement in pain severity of 3.5 cm on the VAS scale, along with a remarkable reduction in the average number of daily pain attacks from 50 to two (a 96% decrease). The average duration of latency was 11 days. The average duration of the treatment’s effectiveness was 14.25 weeks. No adverse reactions were reported.

Patient 2

A 42-year-old female with a 15-year history of trigeminal neuralgia (TN), localized to the V2/V3 divisions on the left hemiface. Medications currently used were carbamazepine. She had a follow-up period of eight years, receiving 34 cycles of treatment with ONA/A during that period. She experienced an average improvement in pain severity of 1.8 cm on the VAS coupled with a decrease in the average number of pain attacks per day from 20 to six (a 70% reduction). The average duration of latency was eight days. The mean duration of treatment effectiveness was 11.9 weeks. Adverse reactions were mild xerostomia and pain.

Patient 3

A 40-year-old female with an eight-year history of trigeminal neuralgia (TN), localized to the V2/V3 divisions on the left hemiface. Medications currently used were amitriptyline. He had a similar eight-year follow-up period during which she received 37 cycles of treatment with ONA/A. She reported a mean improvement in pain severity of 2 cm on the VAS and a decrease in the daily mean number of pain attacks from 12 to 5.6 (a 53.3% reduction). The average duration of latency was 11 days. The mean duration of treatment effectiveness was 10.63 weeks. Adverse reactions were mild xerostomia, pain, oral incontinence, and facial paralysis.

Patient 4

A 49-year-old female with a seven-year history of trigeminal neuralgia (TN), localized to the V3 division on the left hemiface. Medications currently used were oxcarbazepine. She had a follow-up period of five years and received 12 cycles of treatment with ONA/A. She experienced a mean dramatic improvement in pain severity of 8 cm on the VAS and a decrease in the mean number of pain attacks per day from approximately 100 to 10 (a 90% reduction). The average duration of latency was eight days. The mean duration of treatment effectiveness was 24.27 weeks. No adverse reactions were reported.

The mean age of the patients was 46 years (range: 40-53 years), with TN onset occurring after the age of 40 years in all cases. The average time since TN onset was 11 years, and the average follow-up time was 6.25 years. The number of BoNT-A treatments during the follow-up period ranged from a minimum of 12 to a maximum of 37, with a mean of 23.75 injections. This represents a cumulative follow-up period of 25 years, with a total of 95 injections of ONA/A administered. The mean improvement of pain severity on the VAS was 3.72 cm, and the mean reduction in the number of pain attacks per day was 77.32%. The average latency until the onset of treatment effect was 9.5 days, and the average duration of treatment effectiveness was 15.26 weeks. Adverse reactions were exhibited in two patients but with a low degree of severity.

## Discussion

Literature review

Our review identified four studies that met our criteria [19-22]. All of these studies included patients diagnosed with idiopathic TN, as per the ICHD diagnosis criteria, and were refractory to prior treatments at the baseline. It is noteworthy that the definitions of "refractory" varied across these studies, as detailed in Table 4 of Appendices. All patients underwent either MRI or CT scans to exclude the presence of any structural pathology (MRI in the studies by Shehata et al. and Zuniga et al. and MRI/CT in the studies by Wu et al. and Zhang et al.) [19-22].

All participants had previously received preventive medications and, at the baseline, they continued to use medications, such as carbamazepine, gabapentin, or opioids, to alleviate their pain (see Table 5 of Appendices). Throughout the follow-up period, no alterations were made to the prescribed medications, with the exception of a single patient in Wu et al.’s BoNT-A group [19]. In this trial, one patient from the placebo group underwent gamma knife surgery during the follow-up period.

Most exclusion criteria were similar across the four studies. Patients were excluded if they had any underlying medical condition that could increase the risk associated with BoNT-A exposure; an ongoing infection or skin issue at potential injection sites; and women who were pregnant, nursing, or planning a pregnancy or who were unable or unwilling to use a reliable form of contraception during the study. Other exclusion criteria can be found in Table 4 of Appendices.

There was no significant disparity in pain severity and attack frequency between the BoNT-A and placebo cohorts at the initiation of the trials. The baseline demographics and characteristics of each RCT are described in Table 2.

**Table 2:** Baseline demographics, characteristics, studies design, and outcomes NA, not available; PGIC: Patient Global Impression of Change; QoL: quality of life; VAS: Visual Analogue Scale; TN, trigeminal neuralgia; BoNT-A, botulinum neurotoxin type A

Study	Population	Age, years, mean (SD)	Female, N (%)	Time since onset of NT, mean (SD)	Pain intensity, VAS, mean (SD)	Frequency of TN, n/day, mean (SD)	Dose	Duration/primary endpoint, weeks	Primary outcome	Secondary outcome
Wu et al., 2012 [19]	Total (n=42)	58.60 (14.62)	23 (54.76%)	71.07 (80.17) months	6.96 (2.11)	21.15 (17.72)	75 U	1 baseline+12 run-in/12	Pain severity according to 11-point VAS (sic) and pain attack frequency per day	PGIC: 7-point scale; Responders (≥50% reduction in mean pain score)
	BoNT-A (n=22)	59.14 (12.58)	13 (59.09%)	72.05 (78.48) months	7.05 (2.03)	21.71 (22.68)				
	Placebo (n=20)	58.00 (16.91)	10 (50.00%)	70.00 (80.03) months	6.88 (2.25)	20.53 (10.38)				
Shehata et al., 2013 [20]	Total (n=20)	45.95 (10.02)	11 (55%)	5.33 (1.52) years	NA	NA	40-60 U	12/12	Pain severity reduction in the 10 cm VAS score and paroxysms frequency	QoL: 10-point scale; number of weekly acute medications received
	BoNT-A (n=10)	NA	NA	NA	8.3	36.7 (3.13)				
	Placebo (n=10)	NA	NA	NA	8.5	39.20 (3.05)				
Zúniga et al., 2013 [21]	Total (n=36)	NA	17 (47,22%)	NA	NA	NA	50 U	12/12	VAS including the impact on function by the presence or absence of mild, moderate, severe, or disabling pain (sic) during four activities daily	Effects of treatment on the patient’s quality of pain according to a Short Form (36) at baseline and 12 weeks
	BoNT-A (n=20)	64.5 (12.94)	11 (55%)	6.2 (5.01) years	8.85	29.1 (31.36)				
	Placebo (n=16)	66.06 (14.16)	6 (37,5%)	5.2 (3.1) years	8.19	31.06 (31.62)				
Zhang et al., 2014 [22]	Total (n=84)	59.81 (12.30)	44 (55%)	50.96 (46.26)	6.81 (2.12)	NA	25/75 U	1 baseline+8 run-in/8	Pain severity according to 11-point VAS (sic) and pain attack frequency per day	PGIC: 7-point scale
	BoNT-A 25U (n=27)	58.16 (11.54)	15 (60%)	72.64 (76.45)	6.24 (2.13)	NA				
	BoNT-A 75U (n=29)	62.64 (13.32)	16 (57,14%)	71.36 (67.68)	7.18 (2.21)	NA				
	Placebo (n=28)	58.41 (11.74)	13 (48,15%)	91.96 (72.61)	6.96 (1.97)	NA				

The active groups were treated with ONA/A. The control groups received placebo injections consisting of 0.9% saline. The injections were administered via subcutaneous or intradermal routes, and the number of injections and injection sites varied across the studies (see Table 6 of Appendices). The administered dose of ONA/A ranged from a minimum of 25 U in Zhang et al. to a maximum of 75 U in both Wu et al. and Zhang et al. [19,22]. Two studies implemented flexible dosing regimens for BoNT-A (Shehata et al.; Zhang et al.) [20,22]. The follow-up period varied across the studies, spanning from eight weeks to 12 weeks, with follow-up frequencies ranging from weekly to monthly intervals. In the majority of the studies, the primary endpoint was set at 12 weeks, with the exception of Zhang et al., where it was at eight weeks [22]. All studies set pain severity, evaluated through the VAS, and the frequency of pain attacks as primary outcomes. Additionally, two studies included the Patient Global Impression of Change (PGIC) seven-point scale as a secondary outcome measure. To assess treatment efficacy, Wu et al. and Zhang et al. defined responders as those experiencing a ≥50% reduction in the mean pain score from baseline to endpoint [19,22]. Studies design and outcomes are outlined in Table 2.

Primary outcomes

Efficacy of BoNT-A on TN: Reduction of Pain Intensity

All studies reported a statistically significant difference in the reduction of pain intensity between the BoNT-A group and the placebo at the primary endpoint analysis, as detailed in Table 3. In Wu et al. trial, pain intensity in the BoNT-A group significantly decreased after week 2 and this effect was sustained throughout the study, until the 12-week endpoint [19]. The observed difference between groups reached statistical significance (p<0.05) at the primary endpoint. In Shehata et al. trial, pain intensity in the BoNT-A group was reduced from a mean baseline of 8.3 cm to a mean of 1.8 cm at the 12-week post-injection assessment [20]. In contrast, the placebo group presented a decline in VAS from a baseline mean of 8.5 cm to 8.2 cm at the 12-week assessment. The difference between these groups also achieved statistical significance (p<0.0001). In the RCT conducted by Zúñiga et al., VAS scores significantly changed from a baseline mean of 8.85 cm to 4.75 cm in the BoNT-A group at the primary endpoint (12 weeks) [21]. In the placebo group, VAS decreased from a baseline mean of 8.19 cm to 6.94 cm during the same period. The difference between groups was found to be significant (p=0.01). In the study by Zhang et al., the mean VAS scores in both the 25 U and 75 U BoNT-A groups exhibited a significant reduction compared to the placebo group (p<0.017), starting as early as week 1, and the difference was significantly sustained until week 8 (primary endpoint) [22]. No significant difference was observed between the 25 U and 75 U BoNT-A groups throughout the study (p>0.05).

**Table 3:** Studies main results and adverse reactions VAS: Visual Analogue Scale; BoNT-A, botulinum neurotoxin type A; NA: not available

Study	VAS difference vs placebo (p)	VAS change (mean cm)	1st week of difference vs placebo	Frequency of attacks difference vs placebo (p)	Frequency of attacks change (mean)	Adverse reactions
Wu et al., 2012 [19]	p<0.05	NA	2	p<0.05	-19.71 (BoNT), -1.53 (placebo)	Facial asymmetry on the injection area (n=5) (BoNT), edema on the area of injection (n=3) (2 BoNT; 1 placebo)
Shehata et al., 2013 [20]	p<0.0001	-6.5 (BoNT), -0.3 (placebo)	2	p<0.0001	-32.7 (BoNT), -3.1 (placebo)	Facial asymmetry, n=4 (BoNT), hematoma at the site of injection, n=3 (1 BoNT; 2 placebo), itching at the site of injection, N=2 (1 BoNT; 2 placebo), pain at the site of injection, N=2 (1 BoNT; 2 placebo)
Zúniga et al., 2013 [21]	p=0.01	-4.1 (BoNT), -1.25 (placebo)	4-8	p<0.001	-22 (BoNT), -9.81 (placebo)	Hematoma at the site of administration, N=2 (BoNT), slight facial asymmetry due to weakness, N=2 (BoNT)
Zhang et al., 2014 [22]	p<0.017	NA	1	NA	NA	Facial asymmetry in the injection area, N=3 (2 BoNT 25U; 1 BoNT 75U), edema in the injection area N=2 (BoNT 25U)

Efficacy of BoNT-A on TN: Reduction in the Frequency of TN Attacks

In the Wu et al. study, mean attack frequency per day in the BoNT-A group dropped from 21.71 (SD: 22.68) to two attacks/day (90.78% reduction, p<0.05) and in the placebo group from 20.53 (SD: 10.38) to 19 attacks/day (7.45% reduction) at primary endpoint (12 weeks). In Shehata et al., mean paroxysms frequency in the BoNT-A group per day decreased from 36.7 (SD: 3.13) to four attacks/day (SD: 7.12, 89.1% reduction) and from 39.2 (SD: 3.05) to 36.1 (SD: 13.45) in the placebo group (7.9% reduction, p<0.0001) [20]. Similarly, in Zúñiga et al. study, the mean daily attack frequency significantly decreased from 29.1 to 7.1 attacks/day in the BoNT-A group (75.6% reduction, paired t-test, p<0.001) [21]. Conversely, in the placebo group, the mean decreased from 31.06 to 21.25 attacks/day (31.5% reduction) but this reduction was not statistically significant (paired t-test, p=0.19). Finally, in the Zhang et al., the frequency of TN attacks was not reported [22].

Secondary outcomes

Efficacy of BoNT-A on TN: PGIC and Response to Treatment 

In the Wu et al. study, the assessment of PGIC revealed that 77.27% of patients in the BoNT-A group reported their pain symptoms as "much improved" or "very much improved" in contrast to 20.00% in the placebo group (p<0.01) [19]. Additionally, in terms of treatment response, a significantly higher percentage of BoNT-A treated patients (68.18%) responded positively compared to only 15.00% in the placebo group (p<0.01). In the study by Zhang et al., a significant improvement in symptoms was also observed with the use of BoNT-A [22]. In the 25 U group, 75.9% of patients reported being "much improved" or "very much improved" according to the PGIC, compared to 32.1% in the placebo group (p<0.017). Furthermore, the 75 U group also showed a higher proportion of patients reporting “much improved” or “very much improved” (75.9%) compared to the placebo group (p<0.017). Notably, there was no statistically significant difference between the 25 U and the 75 U groups (p>0.05).

Efficacy of BoNT-A on TN: Number of Weekly Acute Medications Received, QoL, and Quality of Pain

In Shehata et al., the BoNT-A group exhibited a significant decrease in the number of weekly acute medication intakes (32; 4.7, mean), and a significant increase in the QoL functioning scale (3.10; 9.0, mean) [20]. In Zúñiga et al., the impact of treatment on the patient's quality of pain, as evaluated using the Short Form (36) (SF36), showed an average score of 90.7 (SD, 22.21) points for subjects treated with BoNT-A [21]. In comparison, for those treated with placebo, the average score was 95.75 (SD, 16.78) points; this 5.05-point difference was not statistically significant (t-test, p=0.46).

Adverse reactions

In the RCT from Wu et al., five patients in the BoNT-A group (75U) experienced facial asymmetry (22.7%), and two patients presented with facial edema (9%) [19]. In the placebo group, one out of the 20 patients also presented with facial edema (5%). All cases of facial asymmetry were resolved by the seventh week, and cases of facial edema were resolved by the seventh day, both with no lasting effects. In the study by Shehata et al., within the BoNT-A group, facial asymmetry was observed in four patients (40%), and three patients (30%) experienced local reactions at the injection site [20]. In the placebo group, the same proportion of four patients (40%) reported local reactions at the injection site. In the Zúñiga et al. study, two out of the 20 patients in the BoNT-A group (50U) presented with hematoma in the injection site (10%), and two presented with facial asymmetry (10%) [21]. Finally, in the Zhang et al. trial, adverse reactions were more prevalent in the 25U BoNT-A group [22]. Specifically, two patients (7.4%) in the BoNT-A 25U group and one patient (3.4%) in the BoNT-A 75U group reported mild facial asymmetry, which was resolved by the six-week assessment. Additionally, two patients (7.4%) in the 25U BoNT-A group presented with facial edema, which resolved within five days. The study's main results and adverse reactions are detailed in Table 3.

According to the American Academy of Neurology and the European Federation of Neurological Societies guidelines, first-line treatments for TN include carbamazepine, oxcarbazepine, baclofen, lamotrigine, phenytoin, topiramate, and pimozide [23,24].

However, these medications are often associated with poor tolerability due to metabolic interactions with other drugs, nervous system depression, dose-related hyponatremia, and potential osteoporosis, particularly in the case of carbamazepine and oxcarbazepine [1].

In refractory cases, surgical intervention may serve as an alternative with a considerable success rate. However, it is associated with a risk of serious complications, including aseptic meningitis, hearing loss, facial palsy, and even death [25].

Micheli et al., first reported a successful treatment of TN with BoNT-A, in 2002, presenting a promising alternative for refractory cases to conventional treatment [26].

The main action of BoNT-A is to inhibit the release of acetylcholine at the neuromuscular junction. The precise mechanism underlying its analgesic effect, however, remains unclear. Growing evidence suggests that the analgesic and anti-inflammatory effects of BoNT-A are mediated through various molecular pathways in both the peripheral nerves and the spinal cord. This clinical observation aligns with animal models that suggest that the analgesic effect of BoNT is, to some extent, mediated by retrograde axonal transport to the spinal cord [10]. One hypothesis suggests that BoNT-A controls pain through its interaction with the SNARE complex, blocking synaptic vesicle fusion and inhibiting the release in the central nervous system of various pain-modulating neurotransmitters, including glutamate, substance P and calcitonin gene-related peptide (CGRRP), hence reducing central sensitization [10,13]. Another proposed mechanism involves the reduction of the expression of transient receptor potential (TRP) channels, specifically TRPA1, TRPV1, and TRPV2, in the spinal trigeminal nucleus, which can directly modulate central sensitization, exerting an antinociceptive function. These channels have been recognized as potential sensors and transducers of pain [27,28]. Yang et al. have also proposed that the antinociceptive effects of BoNT-A may be related to the inhibition of upregulated Nav isoform 1.7 in the Gasserian ganglion [29]. This sodium channel subtype upregulation plays a role in the generation and maintenance of abnormal neuronal electrogenesis and hyperexcitability, potentially explaining the relatively successful usage of sodium channel blockers in TN.

Active groups included in the RCTs of our review were treated with ONA/A and the most commonly used technique involved multiple intradermal and/or subcutaneous injections in the painful area, aligning with our case series. However, injections into the masseter muscle were also employed, particularly in cases with V3 involvement. The doses of BoNT-A have been determined empirically and ranged from 25U to 75U of ONA/A, also similar to doses applied in our case series (ranging from 30U to 60U).

In Shehata et al., there was no significant correlation between the total injected dosages of BoNT-A (ranging from 40U to 60U with a mean of 48U±5.87) and the endpoint VAS (r=0.327, p=0.356) or paroxysms frequency (r=−0.484, P=0.156) [20]. Of note, in the only three-arm study with a direct comparison of two doses (25 U and 75 U), VAS change and proportion of responders were not significantly different between the two active groups from the first to the eighth week [22]. This suggests that using the minimum effective dose may be prudent, which in that particular RCT was 25U of ONA/A. No other study used a lower dose, leaving the question of the lowest effective dose unanswered.

Results from the RCTs included in the literature review favored the use of BoNT-A since the majority of injected patients had a significantly better response compared to the placebo. Overall, attack frequency and pain severity were reduced one to two weeks after injections, and treatment demonstrated efficacy throughout the study follow-up (eight weeks to 12 weeks), as also happened in our case series (mean latency of 9.5 days, mean duration of treatment effectiveness 15.26 weeks). The mean improvement of pain intensity in our case series (3.72 cm, VAS) is comparable to the RCTs included in the review (4.1-6.5 cm, VAS). The reduction in attack frequency ranged from 75.6% to 90.78%, also aligning with our case series (77.32% decrease). Contrary to our case series, studies from our review included secondary outcomes such as the PGIC seven-point scale, number of weekly acute medications received, QoL, and quality of pain with some positive results in these measures. 

Mild and transient adverse reactions were reported in the literature review, such as facial asymmetry, edema, pain, and hematoma, aligning with our case series.

The trials included in our review lack evidence regarding the efficacy of BoNT-A in primary and secondary TN since all patients included had idiopathic TN. Further studies focusing on the effectiveness of BoNT-A for the treatment of primary and secondary TN are needed.

Another critical question arising from the review pertains to the duration of the benefits of BoNT-A injections. None of the studies had a follow-up longer than 12 weeks, potentially limiting the assessment of the analgesic effect, especially when comparing different dosage regimens. Our small case series suggests a therapeutic effect duration ranging from a minimum of 10.63 weeks to a maximum of 24.27 weeks, and an average of 15.26 weeks, with patients routinely receiving treatment injections at regular intervals. Longer-term trials are needed to thoroughly evaluate the optimal dose concerning treatment duration and the frequency of adverse effects.

Notably, the four patients of our case series represent a cumulative follow-up period of 25 years, with a total of 95 injections of ONA/A administered, showing effectiveness throughout the years (range four to eight years), which may point to the safety, and maintained effectiveness of repeated BoNT-A treatments in idiopathic TN, which should be specifically analyzed in future studies.

## Conclusions

Our case series included four patients, which represented a cumulative follow-up period of 25 years, with a total of 95 injections of ONA/A administered. ONA/A doses ranging from 30U to 60U were applied. The mean improvement of pain severity on the VAS was 3.72 cm, and the mean reduction in the number of pain attacks per day was 77.32%. The average latency until the onset of treatment effect was 9.5 days, and the average duration of treatment effectiveness was 15.26 weeks. Adverse reactions were exhibited in two patients but with a low degree of severity. In this group of patients, BoNT-A was effective and safe in the treatment of refractory idiopathic showing effectiveness throughout the years (range four to eight years), which may point to the safety and maintained effectiveness of repeated BoNT-A treatments in idiopathic TN, which should be specifically analyzed in future studies. The review focusing on the use of BoNT-A in the management of TN showed BoNT-A efficacy through a significant reduction in the outcomes of pain intensity and the number of daily attacks. ONA/A doses ranging from 25U to 75U have been employed. The most common adverse effects were facial asymmetry and edema. The optimal dose and the duration of treatment effectiveness remain uncertain, as well as the efficacy and safety of BoNT-A in primary and secondary TN. Further studies with longer follow-up periods are necessary to address these questions and provide a more comprehensive understanding of BoNT-A in the treatment of TN. Based on the available evidence, BoNT-A seems to be effective and safe in the treatment of refractory idiopathic TN for up to 12 weeks. Longer-term trials are needed to thoroughly evaluate the optimal dose concerning treatment duration and the frequency of adverse effects.

## Appendices

**Table 4:** Definition of refractory and exclusion criteria VAS, Visual Analog Scale; TN, trigeminal neuralgia; BoNT-A, botulinum neurotoxin type A

Study	Definition of refractory	Exclusion criteria
Wu et al., 2012 [19]	Failure of recent treatment for TN (pain intensity mean score ≥4; mean attack frequency ≥4 per day)	Use of any agent that might put patients at increased risk if exposed to BoNT-A (e.g., agents that might interfere with neuromuscular function)
Shehata et al., 2013 [20]	Failure to experience at least a 50% reduction in pain intensity quantified by VAS and/or paroxysms frequency, during the last three months, despite the use of proper drugs and dosages, prior to study initiation	Response to medical treatments; possibility of lack of coherence during follow-up
Zúniga et al., 2013 [21]	No satisfactory response to the usual treatment approaches	Changes in the usual medical management 2 months before the study
Zhang et al., 2014 [22]	Pain intensity mean score ≥4; mean attack frequency ≥4 per day; course >4 months	Drugs that interfere with the neuromuscular junction taken within seven days before study entry; significant unstable medical disease or a history of a significant mental disorder; history of substance dependence or abuse

**Table 5:** Medications at baseline/previous surgery NA, not available

Study	Medications at baseline	Previous surgery
Wu et al., 2012 [19]	Carbamazepine (n=36), gabapentine (n=16), opioids (n=3)	NA
Shehata et al., 2013 [20]	Carbamazepine (n=11), oxcarbazepine (n=9), gabapentine (n=10)	Surgical treatment (n=2)
Zúniga et al., 2013 [21]	Carbamazepine (n=31), gabapentin (n=4), amitriptyline (n=1)	Surgical treatment (n=8)
Zhang et al., 2014 [22]	NA	NA

**Table 6:** Injection method and doses

Study	Doses, UI	Dilution	Injections method
Wu et al., 2012 [19]	75	100U/2 mL (5 U/0.1 mL)	Total of 15 points, 5U (0.1 mL) per point, using a “follow the pain” strategy, intradermal and/or submucosal injection according to the patients’ descriptions of painful area.
Shehata et al., 2013 [20]	40-60	100U/2 mL (5 U/0.1 mL)	“Follow the pain” method, especially in trigger zones. Each point was injected subcutaneously with 5U/0.1 mL. Injected doses ranged from 40U (8 injection points) to 60U (12 points) (mean±SD was 48±5.87).
Zúniga et al., 2013 [21]	50-60	50U/1 mL (5 U/0.1 mL)	Subcutaneous injections were given 1 cm apart from one another in the path of the trigeminal branch/branches involved. An extra 10U was given in the masseter muscle if V3 was involved.
Zhang et al., 2014 [22]	25/75	25U or 75U reconstituted with 1 mL	Intradermal and/or submucosal injections were given in 20 points, 0.05 mL/per point, following the area where the pain was experienced according to the patients’ descriptions.

## References

[1] Lambru G, Zakrzewska J, Matharu M (2021). Trigeminal neuralgia: a practical guide. Pract Neurol.

[2] Araya EI, Claudino RF, Piovesan EJ, Chichorro JG (2020). Trigeminal neuralgia: basic and clinical aspects. Curr Neuropharmacol.

[3] Olesen J (2018). Headache classification committee of the international headache society (IHS) the international classification of headache disorders. Cephalalgia.

[4] Nurmikko TJ (2006). Chapter 38 Trigeminal neuralgia and other facial neuralgias. Handbook of Clinical Neurology.

[5] Ostrowski H, Roszak J, Komisarek O (2019). Botulinum toxin type A as an alternative way to treat trigeminal neuralgia: a systematic review. Neurol Neurochir Pol.

[6] Obermann M (2010). Treatment options in trigeminal neuralgia. Ther Adv Neurol Disord.

[7] Piovesan EJ, Teive HG, Kowacs PA (2005). An open study of botulinum-a toxin treatment of trigeminal neuralgia. Neurology.

[8] Allam N, Brasil-Neto JP, Brown G (2005). Injections of botulinum toxin type a produce pain alleviation in intractable trigeminal neuralgia. Clin J Pain.

[9] Gordon D, Finkelstein I, Freund B, Dhawan P (2016). Botulinum toxin type-A in pain management. Pract Pain Manag.

[10] Sandrini G, De Icco R, Tassorelli C, Smania N, Tamburin S (2017). Botulinum neurotoxin type A for the treatment of pain: not just in migraine and trigeminal neuralgia. J Headache Pain.

[11] Camoes-Barbosa A, Neves AF (2016). The analgesic effect of abobotulinum and incobotulinum toxins type A in central poststroke pain: Two case reports. PM&R.

[12] Camoes-Barbosa Camoes-Barbosa, A A (2023). Efficacy of incobotulinumtoxinA for spasticity-associated pain in a series of patients with spasticity of diverse etiologies. Med Res Arch.

[13] Wissel J, Camões-Barbosa A, Comes G, Althaus M, Scheschonka A, Simpson DM (2021). Pain reduction in adults with limb spasticity following treatment with incobotulinumtoxinA: a pooled analysis. Toxins (Basel).

[14] Neves AF, Camões-Barbosa A (2016). Ombro Doloroso Do Hemiplégico: Da Prevenção Ao Tratamento.. Revista Da Sociedade Portuguesa de Medicina Física E de Reabilitação.

[15] Portugal DM, Ferreira EF, Camões-Barbosa A (2021). Botulinum toxin type A therapy for bilateral focal neuropathic pruritus in multiple sclerosis: a case report. Int J Rehabil Res.

[16] Wang A, Jankovic J (1998). Hemifacial spasm: clinical findings and treatment. Muscle Nerve.

[17] Ferreira EF, Camões-Barbosa A (2024). IncobotulinumtoxinA in refractory temporomandibular disorder due to disk dislocation: a prospective study. J Stomatol Oral Maxillofac Surg.

[18] Park HJ, Lee Y, Lee J, Park C, Moon DE (2006). The effects of botulinum toxin A on mechanical and cold allodynia in a rat model of neuropathic pain. Can J Anaesth.

[19] Wu CJ, Lian YJ, Zheng YK (2012). Botulinum toxin type A for the treatment of trigeminal neuralgia: results from a randomized, double-blind, placebo-controlled trial. Cephalalgia.

[20] Shehata HS, El-Tamawy MS, Shalaby NM, Ramzy G (2013). Botulinum toxin-type A: could it be an effective treatment option in intractable trigeminal neuralgia?. J Headache Pain.

[21] Zúñiga C, Piedimonte F, Díaz S, Micheli F (2013). Acute treatment of trigeminal neuralgia with onabotulinum toxin A. Clin Neuropharmacol.

[22] Zhang H, Lian Y, Ma Y, Chen Y, He C, Xie N, Wu C (2014). Two doses of botulinum toxin type A for the treatment of trigeminal neuralgia: observation of therapeutic effect from a randomized, double-blind, placebo-controlled trial. J Headache Pain.

[23] Gronseth G, Cruccu G, Alksne J (2008). Practice parameter: the diagnostic evaluation and treatment of trigeminal neuralgia (an evidence-based review): report of the Quality Standards Subcommittee of the American Academy of Neurology and the European Federation of Neurological Societies. Neurology.

[24] Zakrzewska JM (2010). Medical management of trigeminal neuropathic pains. Expert Opin Pharmacother.

[25] Bond AE, Zada G, Gonzalez AA, Hansen C, Giannotta SL (2010). Operative strategies for minimizing hearing loss and other major complications associated with microvascular decompression for trigeminal neuralgia. World Neurosurg.

[26] Micheli F, Scorticati MC, Raina G (2002). Beneficial effects of botulinum toxin type a for patients with painful tic convulsif. Clin Neuropharmacol.

[27] Aoki KR (2003). Evidence for antinociceptive activity of botulinum toxin type A in pain management. Headache.

[28] Wu C, Xie N, Lian Y (2016). Central antinociceptive activity of peripherally applied botulinum toxin type A in lab rat model of trigeminal neuralgia. Springerplus.

[29] Yang KY, Kim MJ, Ju JS (2016). Antinociceptive effects of botulinum toxin type A on trigeminal neuropathic pain. J Dent Res.

